# Research progress on risk prediction models for the diabetic foot

**DOI:** 10.1007/s00592-025-02505-3

**Published:** 2025-04-19

**Authors:** Haixia Qi, Tao Zhang, Lijie Hou, Qi LI, Ruiping Huang, Lihua Ma

**Affiliations:** 1 School of Nursing, Lanzhou University, Lanzhou, 730011 China; 2 The 940 Hospital of Chinese People’s Liberation Army, Lanzhou, 730050 China; 3 Department of Endocrinology, The First Hospital of Lanzhou University, Lanzhou, 730000 China; 4 Department of Neurology, The First Hospital of Lanzhou University, Lanzhou, 730000 China

**Keywords:** Diabetic foot ulcer, High-risk diabetic foot, Diabetes, Prediction model

## Abstract

**Objective:**

This study aimed to comprehensively review the latest advancements in diabetic foot risk prediction models over the past four years to address the severe challenges posed by diabetic foot ulcers, which are among the leading causes of disability and mortality among diabetic patients. Diabetic foot ulcers are characterized by their complex aetiology, pose a grave threat to life and impose enormous social and economic burdens, thus becoming a critical issue in public health that urgently requires attention. By accurately predicting the risk of diabetic foot and implementing early intervention strategies, this study aimed to reduce its incidence and mortality rates.

**Methods:**

This study employed a systematic review and comprehensive analysis framework, conducted extensive searches of electronic databases (including PubMed, EMBASE, the Cochrane Library, CNKI, etc.) and supplemented these searches with manual literature collection to ensure comprehensive information coverage. During the literature screening and evaluation phase, strict adherence to the predetermined inclusion and exclusion criteria was maintained to guarantee the high quality of the included studies. Further detailed quality assessments, data extraction, and analysis of the selected literature were conducted, with a focus on exploring the construction strategies of risk prediction models, the selection of key variables, the evaluation indicators of model performance, and the validation methods.

**Results:**

By comparing and analysing the differences among studies in terms of methodology, model effectiveness, and practical application potential, this study summarized the development trends of diabetic foot risk prediction models and anticipated future research directions. These findings indicate that with the assistance of advanced diabetic foot risk prediction models, potential risk factors can be identified and addressed early on, thereby effectively reducing the incidence of diabetic foot and significantly improving patients’ quality of life.

**Conclusion:**

This study revealed that diabetic foot risk prediction models have significant effects on accurately identifying risk factors and guiding early interventions, serving as effective tools to reduce the incidence of diabetic foot. Through early identification and intervention, the prognosis and quality of life of patients can be significantly improved, providing important references and guidance for the field of public health.

## Introduction

In recent years, with improvements in living standards and a significant extension of life expectancy, the incidence of diabetes mellitus (DM) has risen sharply, ranking eighth on the global burden of disease list [1]. It profoundly affects hundreds of millions of people worldwide and is often accompanied by various severe complications, posing a considerable challenge to the global health system. Among them, diabetic foot (DF) is a severe and chronic complication of diabetes, and its pathogenesis involves decreased protective function of the lower extremities due to neuropathy, as well as microcirculatory disturbances caused by "macrovascular" and microvascular diseases. Early symptoms may include numbness, chills, and abnormal sensations, and may even lead to infection, osteomyelitis, and necrosis. It is predicted that by 2045, the number of people with diabetes worldwide will reach 700 million [2], of whom 19–34% may develop foot ulcers [3]. Among the over 60 million people with diabetes in China, a quarter face the threat of kidney disease. Moreover, traditional treatments have limited effectiveness, leading to amputation in at least 30–40% of patients. This imposes a heavy burden on both patients and the healthcare system. The current research status of diabetic foot risk prediction models shows a significant trend towards diversification and in-depth development. Currently, the research methods for diabetic foot risk prediction models include mainly statistical methods, machine learning methods, and deep learning methods. These methods collect and analyse various factors related to the risk of diabetic foot to construct prediction models, aiming to achieve accurate prediction of diabetic foot risk.

Statistical methods for diabetic foot risk prediction involve various statistical techniques and models to analyse and predict the risk of developing diabetic foot in diabetic patients. These methods typically include steps such as descriptive statistics, univariate analysis, multivariate analysis, and prediction model construction. Descriptive statistics is the first step in data analysis. It is used to summarize and describe the basic characteristics of the data, such as age, gender, disease duration, blood glucose control, and complications, thereby providing a foundation for subsequent analyses. Univariate analysis is used to explore the association between a single factor and the risk of diabetic foot. Common methods include the chi-square test or t-test. Through this step, factors such as gender, age, disease duration, fasting blood glucose, and glycated hemoglobin that may be related to the risk of diabetic foot can be identified. However, since the development of diabetic foot often involves the interaction of multiple factors, multivariate analysis is required, with logistic regression being the most commonly used method. The model is capable of handling binary or multicategory dependent variables, assessing the strength and direction of the impact of each independent variable on the dependent variable, thereby identifying the independent risk factors for diabetic foot. Finally, based on the identified independent risk factors, a predictive model is constructed to assess the risk of diabetic foot in patients. This process includes model training, validation, and evaluation, with the aim of ensuring the accuracy and reliability of the prediction results.

Machine learning (ML) and deep learning (DL) algorithms have been widely applied in the medical field. Artificial intelligence-based technologies are data driven, meaning that they make decisions on the basis of information in databases and have been used for diagnosing diabetes [4, 5]. Machine learning models such as Support Vector Machine (SVM), Random Forest (RF), and Logistic Regression (LR) can be used. These models are capable of handling various types of clinical data, including patients’ medical history, physiological indicators (such as blood glucose levels, blood pressure, etc.), and laboratory test results [6]. For example, Heald et al. (2019) successfully developed a risk prediction model for diabetic foot ulcers using a univariate logistic regression model with predictors such as HbA1c, age, loss of monofilament sensation, creatinine levels, and history of stroke [7]. In terms of data sources, the application of machine learning models relies extensively on various types of information, among which electronic health records (EHR), medical imaging data, and biomarker test results are the most critical. These data sources provide the models with rich and comprehensive patient information. Some studies have delved deeply into and fully utilized patients’ medical history data, such as the duration of diabetes, detailed records of whether foot ulcers have occurred in the past, and key physiological indicators, such as body mass index (BMI) and glycated hemoglobin (HbA1c) levels, as input features for constructing machine learning models. The selection of these features is aimed at improving the predictive accuracy and reliability of the models, thereby providing strong support for clinical decision-making. Deep learning algorithms are capable of directly extracting features from input data, thereby generating accurate predictions. Deep learning models, such as Convolutional Neural Networks (CNNs), can be used to analyze medical imaging data, such as foot X-rays and ultrasound images, to identify potential risks of diabetic foot ulcers [8]. For example, by analyzing images of the foot vasculature, CNNs can detect vascular lesions, thereby predicting the risk of diabetic foot ulcers [9].

It is crucial to identify patients at high risk of diabetic foot at an early stage and to take corresponding preventive and therapeutic measures. Diabetic foot risk prediction models serve as important tools for assessing patients’ risk of developing diabetic foot. The technical methods can be divided into three main categories: statistical methods, machine learning methods, and deep learning methods. Statistical methods, such as logistic regression and Cox proportional hazards models, establish risk prediction models by analysing the statistical patterns in patient data. Machine learning algorithms, on the other hand, can automatically learn and extract features from large amounts of data to build more complex prediction models. The application of deep learning technology in diabetic foot risk prediction can further improve prediction accuracy. Through model prediction, medical staff can identify high-risk patients for diabetic foot early and take corresponding preventive and therapeutic measures, thereby effectively reducing the incidence of diabetic foot. Additionally, the model can provide a scientific basis for the personalized health management of patients, helping them better control their condition.

## Methods

### Search strategy

When searching for articles, we used the following English search terms: “Diabetic Foot,” “Diabetic Foot Ulcer,” “Prediction Model,” “Risk Prediction Model,” “Risk Prediction,” “Risk Factors,” “Risk Assessment,” “Prognostic Model,” “Risk Score,” and “Prediction Tool.” We conducted computer-based searches in PubMed, the Cochrane Library, Embase, CNKI (China National Knowledge Infrastructure), VIP (China Science and Technology Journal Database), and Wanfang Data for relevant literature on diabetic foot risk prediction models. The searches combined subject terms with keywords. Additionally, we manually searched the references of the included articles. The search period was from December 12, 2019, to September 9, 2024, and the search language was limited to English.

### Literature screening process and results

**Fig. 1:**
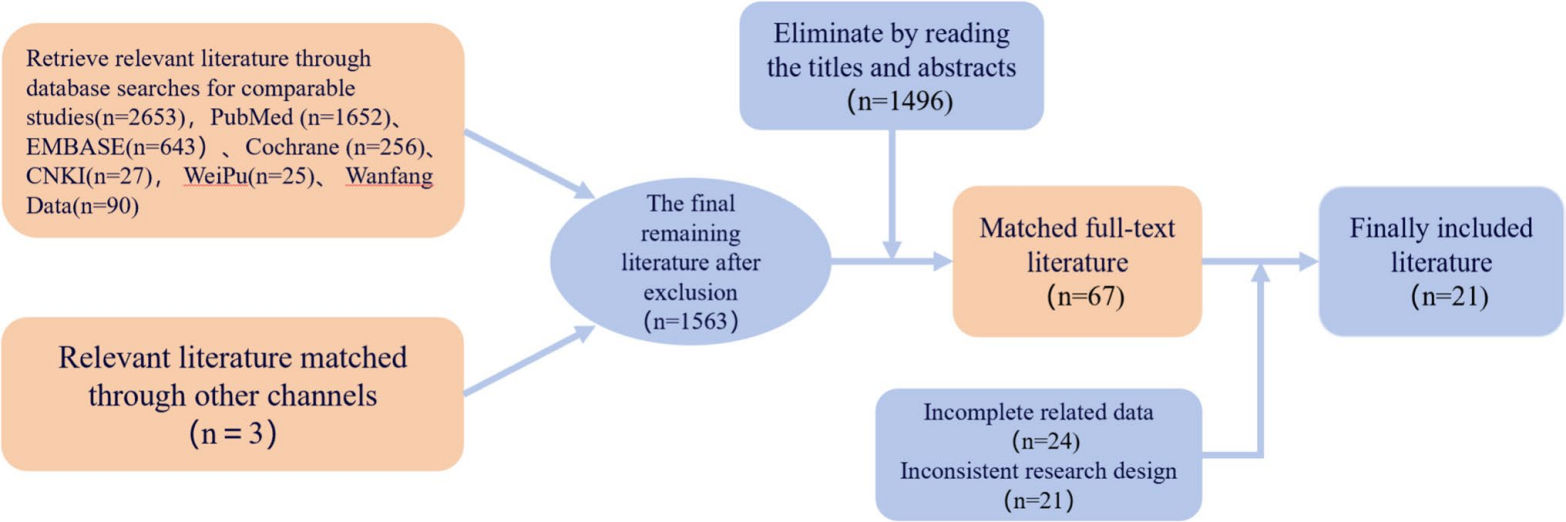
Literature screening flowchart

The literature screening process is shown in Fig. 1. A total of 2,653 relevant articles were retrieved from the PubMed, EMBASE, Cochrane, CNKI, VIP, and Wanfang databases. By focusing on whether the articles contained keywords or phrases such as “diabetic foot,” “risk prediction model,” “predictors,” “risk “assessment”,” etc., we quickly identified articles related to diabetic foot risk prediction models. After excluding irrelevant articles, 1,563 articles remained. By reading the titles and abstracts of each article, we used the exclusion function to remove 1,496 articles, leaving 67 full-text articles. We then read the full texts of the candidate articles one by one, paying attention to whether they described in detail the construction process of the diabetic foot risk prediction model, the selection of predictors, the validation and evaluation of the model, and other key information. On the basis of the research content, purpose, and conclusions of the articles, we assessed their relevance to the research topic and excluded 49 articles. After a combination of initial broad screening and subsequent detailed screening, a total of 21 articles were ultimately included.

### Statistical methods

Statistical methods, as classic data analysis tools, continue to occupy an important position in predicting the risk of diabetic foot. By collecting patients’ clinical data, including age, sex, duration of disease, blood glucose levels, smoking habits, neurovascular disease status, and other factors, researchers have employed statistical methods such as logistic regression and the Cox proportional hazards model to analyse the associations between these factors and the risk of diabetic foot disease. On the basis of these analyses, they constructed prediction models. The common statistical methods and their applications in predicting the risk of diabetic foot have been summarized in Table 1 for further research and application. These models can provide quantitative assessments of the risk of diabetic foot on the basis of individual patient characteristics.

**Table 1:** Statistical methods for predicting diabetic foot

Author	Statistical methods	Purpose	Prediction Result Methods
Iztok Štotl, (2020)[10]	Logistic Regression	It is used to analyze the relationship between multiple independent variables (such as age, gender, duration of illness, blood glucose level, etc.) and the dependent variable (whether diabetic foot occurs), in order to identify the independent risk factors for diabetic foot	Age, duration of diabetes, and blood glucose levels are important risk factors for the occurrence of diabetic foot. Gender indirectly affects the risk of diabetic foot by influencing susceptibility to complications
Peta Ellen Tehan, (2022) [11]	Correlation coefficient	It can clarify the risk of developing diabetic foot and its underlying causes, improving the prediction accuracy and reliability of the model	There is a positive correlation between the risk of diabetic foot and factors such as age, duration of diabetes, blood glucose levels, history of foot ulcers, and history of foot amputations
Fan Hu, (2021) [12]	ROC Curve Analysis	By depicting the relationship between different classification thresholds, it can comprehensively demonstrate the performance of the prediction model and determine the optimal diagnostic threshold for diabetic foot	More accurately distinguishing between patients with diabetic foot and those without diabetic foot
Jun Ho Lee, (2020) [13]	Chi-Square Test	Research has found a correlation between different foot care habits and the occurrence of diabetic foot	There is a strong correlation between poor foot care habits and the occurrence of diabetic foot
Qusai Aljarrah, (2022) [14]	T-test	Study the differences in glycated hemoglo- Aljarrah (2022) [14] bin levels between patients with diabetic foot and those without diabetic foot	There is a significant difference in the mean glycated hemoglobin levels between patients with diabetic foot and those without diabetic "foot".
Lihong Chen, (2023) [15]	Kaplan–Chen Meier Curve	Perform survival analysis on the occurrence time or progression of diabetic foot to describe the survival probabilities of patients in different risk groups	The faster the speed and the greater the magnitude of the decline in the curve, the higher the risk of developing diabetic foot
Zahraa Mansoor, (2022) [16]	Mean and Standard Deviation	It can describe the central tendency of the dataset, reflect the degree of data dispersion, determine weight allocation, and assess the stability of diabetic foot prediction models	variable with a smaller standard deviation implies that the data is more concentrated, and the prediction model is more likely to capture this trend
Flora Mbela Lusendi, (2022) Regression [17]	Cox Proportional Hazards Regression Model	Analyze the relationship between the risk of diabetic foot occurrence and time, while considering the influence of other covariates	A long disease duration is a risk factor for the occurrence of diabetic foot

Statistical methods hold a pioneering position in the field of diabetic foot risk prediction, as they are the first to analyse the clinical data of diabetic patients in depth and identify multiple key factors closely associated with the risk of diabetic foot. On the basis of these core factors, prediction models have been constructed. Among them, a logistic regression model, as a classic representative, can effectively integrate variables such as the patient’s age, gender, duration of disease, blood glucose levels, smoking habits, and neurovascular disease status to accurately assess the patient’s risk of developing diabetic foot. Notably, existing research has revealed a potential association between age and the recurrence of diabetic foot ulcers (DFUs), despite heterogeneity due to varying age stratification criteria [18, 19]. Additionally, smoking, another critical risk factor, has been confirmed by multiple studies to significantly increase the recurrence rate of DFUs, with consistent conclusions [20, 21]. Gender differences in diabetes and its complications have also been widely discussed. In general, the incidence of diabetes is greater in men than in women, especially among middle-aged men [22]. In addition, when diagnosed with type 2 diabetes, male patients tend to have an earlier onset age and a lower BMI [23]. However, female diabetic patients face a greater burden of risk factors at diagnosis, such as higher blood pressure and weight gain, a trend that is particularly prominent among white women and younger women [24, 25].

Diabetic peripheral neuropathy (DPN), a severe complication of type 2 diabetes mellitus (T2DM), exacerbates the risk of ulcers, nontraumatic amputations, and foot infections due to the neurological damage it causes. Moreover, it can lead to long-term disability, imposing heavy economic and psychological burdens on patients [26]. Additionally, research has revealed the intricate interconnections among lower extremity vascular disease, diabetic foot, and diabetic retinopathy, suggesting a trend towards mutual exacerbation among these complications [27]. Correlation coefficients, derived through statistical methods, provide an objective measure of the associations between various factors and the risk of diabetic foot, allowing for more precise identification of high-risk patients for diabetic foot. However, they are data dependent, and the presence of missing, incorrect, or biased data may affect the credibility of the prediction results. The ROC curve graphically displays the sensitivity and specificity of a model at different threshold values, enabling an intuitive understanding of the model’s prediction performance. It can also be used to compare the prediction effects of different models or diagnostic methods. However, the accuracy of a ROC curve depends on the quality and quantity of the samples used. If the samples are biased or insufficient in number, the evaluation results of a ROC curve may be affected. The chi-square test has a broad scope of application and is suitable for various types of data analysis, especially for categorical variable data, such as age, gender, duration of diabetes, complications, and their associations with the risk of diabetic foot. However, the chi-square test can determine only associations and cannot establish causality. The t test is a classic statistical method used to determine whether the difference between the means of two samples is significant. It has high statistical power and can accurately judge whether there is a significant difference between two sets of data, with a wide range of applicability and clear results. However, the t test generally assumes that the data follow a normal distribution or an approximately normal distribution. If the data distribution deviates significantly from a normal distribution, the results of the t test may be affected. The Kaplan‒Meier method constructs survival curves entirely on the basis of empirical data without requiring assumptions about the distribution of the data, making it applicable to various types of data. It visually presents survival rates over time in an easy-to-understand graphical format. However, it cannot directly control or adjust for the influence of confounding factors, which may lead to biased estimates of survival rates. The mean and standard deviation can reflect the central tendency and dispersion of data, providing a more intuitive representation of the data. However, they are susceptible to extreme values and units, which limits their application to a certain extent. The Cox proportional hazards regression model is a semiparametric model that does not require strict assumptions about the distribution of survival times, making it more flexible and widely applicable in practical applications. However, although the Cox proportional hazards regression model is based on the proportional hazards assumption, this assumption may not hold in some cases. For example, when the interaction between the independent variables and time is significant, the hazard ratio may vary over time, leading to potential biases in the model’s results. Additionally, the model is sensitive to outliers and missing values when data are deleted.

### Machine learning methods

Compared with statistical methods, machine learning methods can handle more complex data relationships and uncover hidden risk factors. Common machine learning methods include decision trees, random forests, support vector machines, and gradient boosting trees. These methods automatically learn the nonlinear relationships between risk factors and diabetic foot through training on large amounts of data and constructing efficient prediction models. The common machine learning methods and their applications in diabetic foot risk prediction are summarized in Table 2 for further research and application.

**Table 2:** Machine learning method for predicting diabetic foot

Author	Learning Methods Purpose Prediction Result	Purpose	Prediction result
Zhang J, (2023) [28]	Linear regression	It is possible to calculate the regression coefficients and significance levels of various factors, and identify the independent risk factors for diabetic foot	The duration of diabetes, glycemic control, peripheral neuropathy, and foot deformities are independent risk factors for diabetic foot
Rachita Nanda, (2022)[29]	Random Forest	It can handle a large number of input variables and assess the importance of each variable, enabling comprehensive multi-factor analysis for predicting the risk of diabetic foot	Variables such as blood glucose levels, duration of diabetes, and foot examination results are considered important in predicting the risk of diabetic foot
Shiqi Wang, (2022) [30]	GBDT	The prediction accuracy can be improved by continuously optimizing model parameters, and it can handle nonlinear relationships and interactions	The prediction results from multiple decision trees are accumulated to generate a prediction probability for the risk of diabetic foot occurrence. The higher the probability, the greater the risk
Yu-Long Chen, (2023) [31]	Neural Networks	It can handle large-scale datasets and complex nonlinear relationships, capturing the intricate interactions among multiple risk factors	Input feature data is processed through a nonlinear transformation to obtain a prediction probability value. The higher the probability, the greater the risk
Shichai Hon, (2024) [32]	SVM	Analyze patient data to distinguish between infected and non-infected groups	By analyzing the data, patients are classified into infected and non infected groups
Xiaojin Zhang, (2022)[33]	XGBoost	It can automatically learn and capture complex patterns and features in patient data, employing various optimization techniques to improve prediction accuracy	The input feature data produces an output prediction probability value, where a higher number indicates a greater risk
Zhiyan Fu, (2024) [34]	Ensemble learning method	Combine the prediction results of multiple base learners to improve overall prediction performance	The prediction results from multiple base models are integrated, and the final result is presented in the form of a probability, indicating the risk level of a patient developing diabetic foot

With the rapid development of big data and artificial intelligence technologies, machine learning (ML) has been increasingly applied in the field of diabetic foot ulcer (DFU) risk prediction. Advanced algorithms such as support vector machines (SVMs), random forests, and gradient boosting decision trees (GBDTs) have demonstrated unprecedented potential in DFU risk prediction because of their exceptional data processing and prediction capabilities. For example, researchers have successfully utilized SVMs and backpropagation neural networks (BPNNs) to construct predictive models for the recurrence risk of DFUs. Through comparisons of algorithm performance, it has been confirmed that machine learning models outperform traditional statistical methods in terms of prediction accuracy. However, SVMs have limited processing capabilities for high-dimensional data, and neural networks typically require a large amount of training data to avoid overfitting, with the training process potentially being very time-consuming. Schäfer et al. [10] further utilized machine learning to predict the risk of DFU and amputation in patients with high-risk factors, considering their socioeconomic background and medical history information. A study by Goyal et al. [11] demonstrated the accuracy of machine learning in identifying signs of ischaemia and infection in DFU images. Furthermore, machine learning has been innovatively applied to the analysis of foot thermograms in smartphone applications, enabling early detection of DFU [12]. Thermography technology allows noninvasive, convenient, and easily repeatable foot temperature measurements in diabetic patients [35], facilitating early detection and regular monitoring programs and thereby limiting the incidence of disability-related conditions associated with diabetic foot disease.

Random forest predicts by integrating multiple decision trees, which can significantly improve the prediction accuracy and handle datasets with many features well. However, when the number of decision trees included in the experiment is excessive, the space and time costs required for the training process of the random forest algorithm also increase.

XGBoost, as an efficient machine learning algorithm, excels in processing both structured and unstructured data, achieving higher accuracy than other algorithms do. It can also handle data issues such as missing values and outliers. However, XGBoost has many parameters that need to be tuned, requiring considerable time and effort to find the optimal parameter combination. Additionally, it is susceptible to the influence of outliers or noisy data. Ensemble learning can improve prediction accuracy by combining the predictions of multiple learners, and it has a certain degree of tolerance for errors and outliers from individual learners. However, in practical applications, it requires training multiple learners, resulting in higher computational complexity.

Notably, machine learning is not a blind computation process but rather a process of intelligently extracting reasonable answers on the basis of specific inputs. In summary, ML algorithms create a mathematical model that maps “features” (i.e., observational variables) to “labels” (i.e., outcome variables) [13]. In this process, precise definitions of complex features and labels are crucial. This model not only facilitates early identification of complications such as severe infections but also provides a solid foundation for improving the clinical outcomes and prognosis of patients with diabetic foot ulcers (DFUs).

### Deep learning methods

The deep learning method, as a cutting-edge branch of machine learning, has also demonstrated great potential in predicting the risk of diabetic foot disease. Deep learning is capable of automatically extracting high-level feature representations from raw data, thereby more accurately capturing the complex relationships between risk factors and diabetic foot. Common deep learning methods include convolutional neural networks (CNNs) and recurrent neural networks (RNNs). These methods have unique advantages in processing time series data, image data, and other types of information, providing new insights for predicting the risk of diabetic foot. A summary of common deep learning methods and their applications in predicting the risk of diabetic foot is presented in Table 3 for further research and application [11, 36–40].

**Table 3:** Deep learning method for predicting diabetic foot

Author	Deep learning method	Purpose	Prediction Result
Amith Khandakar, 2021 [11]	Convolutional Neural Network	Used in the automatic identification and classification of diabetic foot ulcer (DFU) images, it predicts risks by analyzing wound images	Predicting risks through wound image analysis
V Sathya Preiya, (2 023) [36]	Recurrent Neural Network	It can capture long-term dependencies in the data, enabling more accurate prediction of the risk of diabetic foot	By inputting data, it outputs a predicted probability value, where a higher probability indicates a higher risk
Yufan He, (2021) [37]	Autoencoder	Used for feature extraction, aiding in the discovery of potential variables related to risk	By inputting data, a prediction probability is obtained, and the higher the probability, the greater the risk
Manu Goyal, (2020) [38]	Deep Learning Ensemble Methods	Improving overall prediction performance by combining the prediction results of different models	Through processes such as data. preprocessing, model training, and ensemble strategies, a prediction probability value is obtained, and the higher the probability, the greater the risk
Jing Zhao, (2022) [39]	Generative Adversarial Networks, GANs	Used for generating high quality simulated data to make the model’s predictions of diabetic foot more accurate	Through data generation and feature enhancement, a prediction probability value is obtained, which categorizes patients into high-risk and low-risk groups
Maheswari D, (2024) [40]	Attention Mechanisms	It can improve prediction accuracy, enhance model interpretability, and promote the enhancement of diabetic foot prediction model performance	Different risk characteristics are assigned weights, and a risk probability is obtained based on these weights. The higher the probability, the greater the risk

Deep learning is an important branch of machine learning that uses artificial neural networks and large datasets to solve computationally complex problems. By mimicking the structure and function of the human brain’s neural networks, it achieves automatic learning and feature extraction from complex data. In the diagnosis of diabetic foot, deep learning models can automatically extract valuable features from images, signals, and other data related to diabetic foot, thereby enabling accurate classification and prediction. This study provides new ideas and methods for the diagnosis and staging of diabetic foot disease. By constructing deep learning models based on images and PPG signals, early screening and accurate staging of the diabetic foot can be achieved, providing timely and effective treatment recommendations for patients. Research has shown that the use of convolutional neural network (CNN) models is more effective in identifying ischaemia and infection. Compared with manual machine learning algorithms, the integrated CNN deep learning algorithm performs better in both classification tasks, with an accuracy rate of 90% for ischaemia classification and 73% for infection classification [11]. However, CNNs have high requirements for data quality and consume significant computational resources, which can be a challenge for resource-constrained environments. The integration of attention mechanisms and deep learning methods can improve model performance and enhance model interpretability. However, in practical applications, they have high computational complexity and complex parameter tuning, requiring longer training times and more computational resources.

Generative adversarial networks (GANs) possess powerful data generation capabilities and flexible model structures, which can improve model accuracy when applied. However, their drawbacks include the difficulty of training and high computational resource requirements. Autoencoders can extract features highly related to the risk of diabetic foot from complex medical data, improving the accuracy and efficiency of predictions. They can also detect anomalies in the input data. However, autoencoders tend to perform poorly when testing data.

## Richness of predictive factors

The predictive factors of the diabetes foot risk prediction model cover multiple aspects, including patients’ basic information (such as age, sex, BMI, etc.), duration of diabetes and complications (such as retinopathy, peripheral neuropathy, etc.), foot examinations (such as dorsalis pedis artery pulsation, abnormal changes in foot skin, etc.), biomechanical parameters (such as plantar pressure, plantar soft tissue stiffness, etc.), and laboratory tests (such as glycated haemoglobin, ankle‒brachial index, etc.). The comprehensive application of these predictive factors enables the model to assess patients’ risk of developing diabetic foot more comprehensively.

### Factors related to underlying diseases

Duration of diabetes: The longer the duration of diabetes is, the greater the risk of developing diabetic foot [41]. With a longer duration of diabetes, the likelihood of distal lower limb vascular pathology increases, and conditions such as atherosclerosis in the lower limb blood vessels can lead to inadequate blood supply to the lower limbs. Long-term diabetes may also affect the dilation and contraction functions of blood vessels, increasing the susceptibility of lower limb blood vessels to damage when subjected to external pressure. A prolonged duration of the disease can also exacerbate distal lower limb neuropathy, reducing or even eliminating patients’ sensitivity to sensations such as pain and temperature. Motor neuropathy may lead to issues such as atrophy of foot muscles and decreased muscle strength, further affecting patients’ ability to walk and stand and increasing the risk of developing diabetic foot.

Blood glucose control status: Patients with poor blood glucose control, such as those with elevated fasting plasma glucose (FPG) levels and glycated haemoglobin (HbA1c) levels [42], are more prone to neuropathy and angiopathy, thereby increasing the risk of diabetic foot. When diabetic patients have poor blood glucose control, hyperglycaemia for an extended period can cause damage to blood vessels and nerves. This damage can lead to lower limb vascular and neuropathic complications, thereby increasing the risk of developing diabetic foot. Hyperglycaemia also provides favourable conditions for bacterial growth, increasing the risk of foot infections.

### Factors related to vascular pathology

Peripheral Arterial Disease: Peripheral arterial disease is an important risk factor for diabetic foot, with a hazard ratio as high as 3.43 [43]. Vascular pathology leads to impaired blood circulation in the lower limbs, affecting wound healing. Macrovascular disease can cause insufficient blood supply to the lower limbs, triggering ischaemic pain in the limbs. In severe cases, it can lead to intermittent claudication, resting pain, and even limb necrosis. Vascular stenosis and occlusion not only affect the blood supply to the feet but also increase the risk of lower limb infections, ulcers, and gangrene.

### Factors related to neuropathy

Peripheral neuropathy: Peripheral neuropathy is another important risk factor for diabetic foot, with a hazard ratio of 2.09 [44]. Neuropathy leads to a decrease or loss of sensation in the feet, making patients susceptible to foot injuries from trauma, burns, etc. When the feet are injured, patients may be unable to detect it in a timely manner, leading to delayed treatment, an increased risk of infection, and ultimately triggering diabetic foot. The autonomic nerves are responsible for regulating skin temperature, humidity, and sweat secretion. When the autonomic nerves are damaged, these functions become abnormal, leading to issues such as dry and cracked skin, which further increases the risk of foot infections.

### Factors related to other complications

Retinopathy: Retinopathy is associated with diabetic foot, with a hazard ratio as high as 6.42 [45]. Retinopathy may reflect the status of systemic microvascular pathology and increase the risk of diabetic foot. The occurrence of retinopathy indicates the presence of microvascular pathology in the patient’s body, which may also affect the blood vessels in the lower limbs.

Nephropathy: Conditions such as microalbuminuria are also among the risk factors for diabetic foot. Patients with kidney disease often have systemic vascular pathology and metabolic disturbances, which increase the risk of foot complications [43]. These patients typically experience vascular pathology that leads to poor blood circulation in the lower limbs. The occurrence of diabetic foot is closely related to impaired blood circulation in the lower limbs; thus, patients with kidney disease are more prone to developing diabetic foot. Kidney disease can also exacerbate neuropathy in diabetic patients, especially neuropathy in the lower limbs. Neuropathy reduces or eliminates patients’ sensitivity to sensations such as pain and temperature, thereby increasing the risk of foot injuries and infections and affecting the healing of the diabetic foot.

### Lifestyle habits and environmental factors

Smoking: Smoking is an independent risk factor for diabetic foot, and smokers have a significantly increased risk of developing diabetic foot [46]. Smoking exacerbates vascular and neuropathic complications, affecting the blood supply and nerve sensation in the lower limbs and increasing the susceptibility of the feet to injury and infection.

Improper foot care: Improper foot care, such as wearing unsuitable footwear and socks and improper nail trimming [47], can lead to foot injuries and infections. Improper foot care can also accelerate the progression of diabetic foot pathology, exacerbate existing symptoms, and accelerate the process of diabetic foot deterioration.

## Rigorousness of model evaluation and validation

**Fig. 2:**
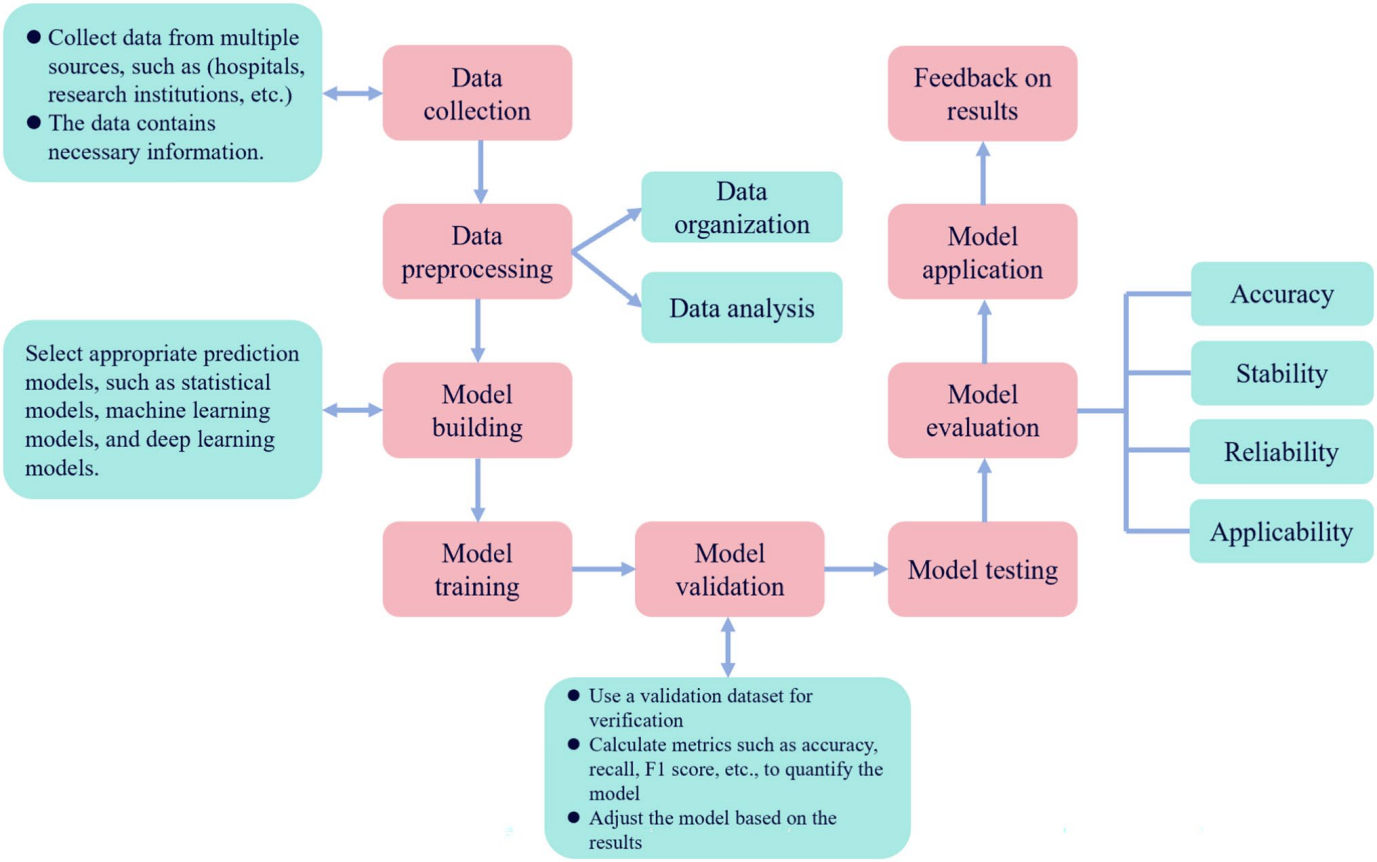
Overview diagram of the research process

The overall construction of a risk prediction model for diabetic foot disease is a complex and systematic process aimed at assessing the risk of diabetic patients developing diabetic foot disease through statistical analysis and machine learning techniques. The specific process is shown in Fig. 2. The process begins with data collection from various sources, such as hospitals and medical institutions, ensuring that the collected data contain all of the necessary information. Next, the data are organized and analysed. On the basis of these data, an appropriate prediction model (statistical model, machine learning model, or deep learning model) is selected. The selected algorithm is then trained on the training dataset to obtain a preliminary prediction model. This model is further validated via a validation dataset, with metrics such as accuracy and recall being calculated to quantify the model’s performance. Adjustments are made to the model on the basis of these results. Model testing and evaluation are subsequently conducted to assess the model’s accuracy, stability, reliability, and applicability. Finally, the model is applied in practical settings (such as communities and hospitals), and feedback is obtained on the basis of the results.

### Selection and processing of predictive factors

The selection of predictive factors should be based on scientific research and clinical experience, covering multiple aspects of the occurrence and development of diabetic foot, such as underlying diseases, vascular pathology, neuropathy, lifestyle habits, and environmental factors. The processing of continuous and categorical variables needs to be scientific and reasonable, maintaining the continuity and authenticity of the data to avoid distortion or oversimplification.

### Selection of modelling methods

Comprehensive data related to the risk of diabetic foot, including but not limited to the patient’s age, sex, duration of diabetes, blood glucose control status, foot examination results, lifestyle habits (such as smoking and alcohol consumption), and complications (such as kidney disease and retinopathy), were collected. Data preprocessing, which includes handling missing values, detecting outliers, data normalization, etc., is necessary before modelling to ensure the quality and consistency of the data. During the modelling process, it is necessary to screen out features closely related to the risk of diabetic foot to improve the prediction accuracy and efficiency of the model. Choosing the right model is crucial for successful modelling. Each model has its own characteristics and applicable scenarios, and the choice should be based on the characteristics of the data and prediction requirements.

### Evaluation metrics for model performance

The area under the receiver operating characteristic (ROC) curve (AUC) [48] was used as the primary metric to evaluate the predictive performance of the model. The closer the AUC value is to 1, the better the predictive performance of the model. Additionally, other evaluation metrics, such as sensitivity, specificity, positive predictive value, and negative predictive value, can also be considered to comprehensively assess the model’s predictive performance.

### Rigorousness of model validation

1. Internal Validation: Internal validation of the model is conducted through data splitting methods (such as the bootstrap method) [49] or cross-validation methods (such as K-fold cross-validation) [50] to assess the stability and reliability of the model on the training dataset. Internal validation helps to identify potential overfitting or underfitting issues in the model during the training process and allows corresponding adjustments and optimizations to be made.
2. External Validation: External validation of the model is conducted via a new dataset that is independent of the training dataset to assess the model’s prediction accuracy and generalizability to unknown data. External validation is a crucial step in assessing the practical application value of the model, as it ensures that the model maintains a stable predictive performance across different populations and environments.
3. Sensitivity Analysis and Robustness Testing: Sensitivity analysis is conducted on the key predictive factors in the model to assess their degree of influence on the model’s prediction results. Robustness testing is performed to evaluate the stability and reliability of the model under different conditions, such as missing data and outlier handling.

## Results

### Statistical methods

A logistic regression model, as a classic method, can integrate information from multiple variables (up to more than 10), providing personalized risk assessments for patients with a prediction accuracy rate of over 80%. Key risk factors, such as age (especially for patients over 60 years old) and smoking, have been extensively studied and confirmed to be closely associated with the risk of diabetic foot ulceration (DFU). Smoking can increase the recurrence rate of DFU by approximately 50%. Furthermore, sex differences are also significant for diabetes and its complications, with a higher incidence rate among males than females. However, females face more risk factors when they are diagnosed.

### Machine learning methods

The ability of machine learning to predict the risk of diabetic foot ulcers (DFUs) is becoming increasingly widespread, and its powerful data processing capabilities enable us to uncover more complex and hidden risk factors. Compared with traditional statistical methods, machine learning algorithms such as decision trees, random forests, support vector machines (SVMs), and gradient boosting decision trees (GBDTs) can automatically learn the nonlinear relationships between risk factors and diabetic foot ulcers (DFUs) through extensive data training, enabling the construction of efficient prediction models.

### Deep learning methods

Deep learning has tremendous potential in predicting the risk of diabetic foot ulcers (DFUs). By simulating the neural networks of the human brain, it extracts high-level features from data, accurately capturing the connections between risk factors and DFUs. Common methods such as convolutional neural networks (CNNs) and recurrent neural networks (RNNs) have advantages in processing specific data, enabling automatic feature extraction and precise predictions, which aid in diagnosis and staging. Deep learning models based on images and PPG signals can perform early screening and accurate staging of diabetic foot ulcers (DFUs). Convolutional neural networks (CNNs) excel in identifying ischaemia and infection, but they have high requirements for data quality and computational resources.

## Discussion

In this systematic review and meta-analysis, we conducted a thorough examination and integration of a vast array of literature, deeply exploring the key factors influencing the development of diabetic foot ulcers (DFUs) as well as the effectiveness of preventive intervention measures. Using statistical methods, we identified the significant roles of variables such as age, gender, duration of disease, blood glucose levels, smoking habits, and neurovascular pathology in the occurrence and progression of DFU. These findings not only provide a scientific basis for our understanding of the pathogenesis of DFU but also offer important references for formulating targeted prevention and treatment strategies. Furthermore, we have also attempted to apply machine learning methods in predicting and assessing the risk of DFU. By training machine learning models, we successfully identified DFU patients with high-risk factors and validated the accuracy of machine learning in recognizing signs of ischaemia and infection in DFU images. The deep learning model integrates patients’ historical data, images, and textual information to accurately identify the risk of foot complications, enabling early screening of diabetic foot ulcers. These discoveries not only expand the application scope of machine learning and deep learning in the medical field, but also provide new technological means for the early diagnosis and treatment of DFU. However, when exploring the effectiveness of comprehensive care in reducing the risk of DFU, we encountered the challenge of low certainty evidence. This suggests that in future work, we need to integrate data from multiple studies through systematic reviews and meta-analyses, expand the sample size to enhance the certainty of evidence; design rigorous randomized controlled trials to ensure comparability between the intervention and control groups, effectively reducing bias; conduct long-term follow-ups to assess the sustained effects of comprehensive nursing; and simultaneously incorporate real-world data to address the limitations of clinical trials, comprehensively validating the effectiveness of comprehensive nursing. Deep learning models are capable of efficiently processing complex clinical data and have demonstrated excellent performance in early screening for diseases such as diabetic foot ulcers, assisting doctors in identifying risks earlier. However, their limited interpretability and lack of transparency in the decision-making process make it difficult for doctors to understand the reasoning logic of the models, which may affect their trust in the results. In future work, a comprehensive evaluation of the models should be conducted through a combination of external validation and clinical validation. Specifically, the generalization ability of the models should be tested on independent datasets to ensure their reliability across different populations and scenarios, and the model predictions should be compared with clinicians’ diagnoses to assess consistency and accuracy. Additionally, interpretability techniques can be leveraged to reveal the decision-making basis of the models and help doctors understand the key features. In the initial stages, the models can be used as auxiliary tools, with doctors retaining the final decision-making authority, to gradually build trust. Doctors should critically assess the model results in combination with clinical experience, while actively exploring efficient modes of collaboration to improve the overall quality of medical decision-making.

## Conclusions

In summary, this systematic review and analysis not only uncovered the key factors influencing the development of DFU but also explored the potential application of machine learning in predicting DFU risk and the effectiveness of comprehensive care in reducing DFU risk. Although we have made significant progress in some areas, there are still issues and challenges that need to be addressed. In the future, the development of diabetic foot ulcer (DFU) risk prediction models will focus more on model optimization and personalization. By continuously collecting and analysing new clinical data, we anticipate the emergence of more accurate and personalized prediction models, which will assist doctors in formulating more precise prevention and treatment plans, thereby increasing the effectiveness of DFU prevention and treatment success rates. Moreover, research on DFU prevention intervention measures, especially comprehensive care methods for high-risk populations and interventions specifically targeting populations with low to moderate ulcer risk, is needed. By designing and conducting high-quality randomized controlled trials, we can provide a more solid scientific basis and technical support for the prevention and treatment of DFU. Finally, we call on the medical community and all sectors of society to work together, strengthen their emphasis and support for DFU prevention and control efforts, and jointly promote progress in diabetic foot prevention and treatment.
